# *Dipterocarpus tuberculatus* as a promising anti-obesity treatment in *Lep* knockout mice

**DOI:** 10.3389/fendo.2023.1167285

**Published:** 2023-05-26

**Authors:** Yu Jeong Roh, Su Jin Lee, Ji Eun Kim, You Jeong Jin, Ayun Seol, Hee Jin Song, Jumin Park, So Hae Park, Bounleuane Douangdeuane, Onevilay Souliya, Sun Il Choi, Dae Youn Hwang

**Affiliations:** ^1^ Department of Biomaterials Science (BK21 FOUR Program)/Life and Industry Convergence Research Institute, College of Natural Resources and Life Science, Pusan National University, Miryang, Republic of Korea; ^2^ Department of Food Science and Nutrition, College of Human Ecology, Pusan National University, Busan, Republic of Korea; ^3^ Institute of Traditional Medicine, Ministry of Health, Vientiane, Laos; ^4^ Henan Key Laboratory of Brain Targeted Bio-Nanomedicine, School of Life Sciences & School of Pharmacy, Henan University, Kaifeng, Henan, China; ^5^ Longevity Wellbeing Research Center/Laboratory Animals Resources Center, College of Natural Resources and Life Science, Pusan National University, Miryang, Republic of Korea

**Keywords:** anti-obesity, *D. tuberculatus*, lipogenesis, lipolysis, inflammasome, β-oxidation

## Abstract

**Introduction:**

The therapeutic effects and mechanisms of *Dipterocarpus tuberculatus* (*D. tuberculatus*) extracts have been examined concerning inflammation, photoaging, and gastritis; however, their effect on obesity is still being investigated.

**Methods:**

We administered a methanol extract of *D. tuberculatus* (MED) orally to *Lep* knockout (KO) mice for 4 weeks to investigate the therapeutic effects on obesity, weight gain, fat accumulation, lipid metabolism, inflammatory response, and β-oxidation.

**Results:**

In *Lep* KO mice, MED significantly reduced weight gains, food intake, and total cholesterol and glyceride levels. Similar reductions in fat weights and adipocyte sizes were also observed. Furthermore, MED treatment reduced liver weight, lipid droplet numbers, the expressions of adipogenesis and lipogenesis-related genes, and the expressions of lipolysis regulators in liver tissues. Moreover, the iNOS-mediated COX-2 induction pathway, the inflammasome pathway, and inflammatory cytokine levels were reduced, but β-oxidation was increased, in the livers of MED-treated *Lep* KO mice.

**Conclusion:**

The results of this study suggest that MED ameliorates obesity and has considerable potential as an anti-obesity treatment.

## 1 Introduction

Southeast Asia has a large distribution of the medicinal herb *Dipterocarpus tuberculatus* (*D. tuberculatus*), particularly in Bangladesh, Thailand, Cambodia, Laos, Myanmar, and Vietnam (1). The parts of *D. tuberculatus* continue to be used as traditional medicines; for example, its leaf gum is used as an anti-venom, and its roots have anti-inflammatory and anti-dysenteric effects (1). Consequently, the therapeutic effects and underlying mechanisms of *D. tuberculatus* have been investigated for their effects on anti-inflammatory response, anti-photoaging, and promotion of osseointegration. An ethanolic extract of *D. tuberculatus* strongly suppressed macrophage-mediated inflammatory responses *in vitro* and *in vivo* downregulated PDK1/NK-κB signaling pathway and suppressed EtOH/HCl-induced acute gastric lesions (2). Moreover, it inhibited lipopolysaccharide (LPS) induced activator protein-1 (AP-1)-mediated inflammatory reaction in macrophages and protected against acute liver injury (3). Furthermore, a methanolic extract of *D. tuberculatus* (MED) exhibited significant anti-photoaging effects by inhibiting apoptosis, cell cycle arrest, age-related extracellular matrix structural changes, and inflammation in UV-irradiated cells and nude mice (4), protective effects in blue light-induced retinal degeneration (5), and was also found to stimulate focal cell adhesion via the MCL2/FAK/Akt signaling pathway (6) and induce the proliferation and adhesion of osteoblasts, which led to bone formation and regeneration in tibia implantation models when coated on the surfaces of titanium plates (7). However, the effects of *D. tuberculatus* on obesity have not been elucidated.

The greatest healthcare challenge in our generation is regulating excessive body fat (8). Over the past 50 years, the global obesity rate has more than tripled, according to World Health Organization (9, 10). Obesity goes far beyond just being overweight; it promotes alarming rates of diabetes, which is associated with an increased risk of type I diabetes, hypertension, hyperlipidemia, cardiovascular disease, and certain cancers, which are major causes of premature death (11, 12). Numerous factors contribute to the onset and progression of obesity and its complications. Among these, hormones such as leptin, adiponectin, and visfatin play a significant role in the regulation of food consumption and energy expenditure by hormones such as leptin, adiponectin, and visfatin are important factors (13–18). Moreover, high serum fatty acid (FA) and triglyceride (TG) levels trigger fat accumulation in adipocytes and lead to oxidative stress, hypertriglyceridemia, lipotoxicity, diabetes, and various metabolic syndromes (19). Thus, reductions in circulating and stored fat levels, lipase inhibition, appetite suppression, energy expenditure stimulation, adipocyte differentiation inhibition, and lipolysis activation are considered key anti-obesity strategies (8, 20) and have been widely utilized to investigate the therapeutic effects of natural products *in vitro* and *in vivo*.

In this study, we investigated the anti-obesity effect and mechanism of action of a MED and its mechanism of action in an obese mouse model. Our findings provide the first evidence that the anti-obesity effects of MED are attributable to the regulation of multiple targets associated with lipid metabolism, including lipid accumulation, lipolysis, the inflammasome, cytokine expression, and the expressions of related signaling molecules in *Lep* knockout mice.

## 2 Materials and methods

### 2.1 Preparation and methanol extraction

The International Biological Material Research Center of the Korea Research Institutes of Bioscience and Biotechnology (Daejeon, Republic of Korea) provided a lyophilized sample of MED (FBM 213-075). Briefly, dried stem powder of *D. tuberculatus* was mixed with methanol in a ratio of 1:10 (wt/vol). The mixture was sonicated for 15 min and incubated for 2 h at room temperature. The process was repeated 10 times daily for 3 days and filtered using a 0.4 µm filter. The extracted solution was concentrated using a rotary evaporator (N = 1000 SWD, EYELA, Bohemia, NY, USA) and lyophilized using a speed vacuum concentrator (Modulspin 40, Biotron Co., Marysville, WA, USA). The MED obtained was dissolved in 1×PBS buffer to the required concentrations for administration to mice.

### 2.2 Liquid chromatography-electrospray ionization mass spectrometry analysis

The bioactive compounds in MED were identified as previously described by Lee et al. (4). Liquid chromatography-mass spectrometry (LC-MS) analysis was performed with a BEH C18 Column (2.1 × 100 mm, 1.7 μm) (Waters, Milford, MA, USA) using an Agilent 1290 Infinity HPLC system (Agilent Technologies, Waldbronn, Germany). Mass spectra were obtained in the negative mode electrospray ionization (ESI) using MassHunter software (Agilent Technologies).

### 2.3 Experiments on *Lep* KO mice

*Lep* KO mice are a useful model for metabolic studies as they display hyperphagia, early-onset obesity, and symptoms of metabolic disease, including increased fatty acid synthesis in fat and liver, impaired glucose tolerance, insulin sensitivity, and hepatic steatosis (18, 21).

The animal experimental protocol for *Lep* KO mice (C57BL/6-*Lep* ^em1Shwl^/Korl) was approved by the Institutional Animal Care and Use Committee of Pusan National University (PNU- Institutional Animal Care and Use Committee (IACUC); Approval Number PNU-2021-0072). *Lep* KO and wild-type (WT, C57BL/6/Korl) mice were maintained at the Pusan National University-Laboratory Animal Resources Center, which is accredited by the Korea Food and Drug Administration (Accredited Unit Number: 000231) and the Association for the Assessment and Accreditation of Laboratory Animal Care International (Accredited Unit Number: 001525). Four-week-old *Lep* KO (n = 21) and WT (n = 7) mice were kindly provided by the Department of Laboratory Animal Resources at the National Institute of Food and Drug Safety Evaluation (NIFDS, Chungju, Korea). Animals were allowed *ad libitum* access to filtered water and a standard irradiated chow diet (Samtako BioKorea Co., Osan, Korea) (crude protein 20%, crude fat 4.5%, crude fiber 6%, crude ash 7%, calcium 0.5%, Phosphorus 1%) throughout the experimental period. Mice were maintained under specific pathogen-free (SPF) conditions at 23 ± 2°C and 50 ± 10% relative humidity (RH) under a daily light cycle (lights on at 08:00 h and off at 20:00 h). The genotype of *Lep* KO mice was identified using DNA-PCR, as previously reported in the literature (22, 23).

Mice were classified into two groups: WT mice (the WT group, n = 7) and obese mice (the *Lep* KO group, n = 21). Mice in the *Lep* KO group were divided into the following three groups; (1) the 1x PBS administrated group (the Vehicle-treated Lep KO group, n = 7), (2) the 100 mg/kg of MED administrated group (the LMED-treated *Lep* KO group, n = 7), and (3) the 200 mg/kg of MED administrated group (the HMED-treated *Lep* KO group, n = 7). The dosages for MED treatment were decided based on results from previous research using identical extract (4, 5). The same volume of Vehicle (PBS) or MED solution was administered orally daily for 4 weeks. Mice were euthanized with CO_2_ at 24 h following final treatments after a 12 h fasting. Tissue samples and sera were acquired and stored in Eppendorf tubes at −70°C until required for the assay.

### 2.4 Measurement of body and organ weights

Mouse weights were measured daily at 10:00 a.m. using an electronic balance (Mettler Toledo, Greifensee, Switzerland), according to KFDA guidelines. In addition, weights of livers and abdominal fat were obtained after sacrifice using the same method.

### 2.5 Serum biochemical analysis

Blood samples from an abdominal vein were incubated for 30 min at room temperature (RT) in serum-separating tubes (BD Containers, Franklin Lakes, NJ, USA). Serum samples were obtained by centrifugation at 1,500 ×g for 15 min, and serum triglyceride (TG), total cholesterol (TC), high-density lipoprotein-cholesterol (HDL-C), and low-density lipoprotein-cholesterol (LDL-C) levels were determined using an BS-120 Automatic Chemical Analyzer (Mindray, Shenzhen, China). All assays were conducted in duplicate using a fresh serum.

### 2.6 Histopathological analysis

Liver and fat tissues were fixed overnight in 10% neutral buffered formalin (pH 6.8), embedded in paraffin wax, and sectioned (4 µm) using a Leica microtome (Leica Microsystems, Bannockburn, IL, USA). Sections were collected on glass slides, deparaffinized with xylene (DaeJung, Gyeonggi-do, Korea), and rehydrated with graded ethanol (100 to 70%) and distilled water. Sections were then stained with hematoxylin and eosin (H&E; Sigma Aldrich Co., St. Louis, MO, USA), and numbers of lipid droplets in liver tissues were counted using the Leica Application Suite (Leica Microsystems, Heerbrugg, Switzerland). Also, areas of adipocytes in fat sections were measured using Image J 1.52a (NIH, Bethesda, ML, USA).

### 2.7 Quantitative reverse transcription polymerase chain reaction analysis

The mRNA levels of PPARγ, C/EBPα, aP2, FAS, adenylyl cyclase (AC), PDE4, CPT1, PPARα, NF‐κB, TNF‐α, IL‐6, and IL‐1β in liver tissues were measured by RT‐qPCR, as previously described (24). Briefly, total RNA in liver tissues was purified using RNAzol (TelTest Inc., Friendswood, TX, USA) and quantified using a NanoDrop system (Biospecnano, Shimadzu Biotech, Kyoto, Japan), complementary DNA (cDNA) was synthesized using a mixture of total RNA (5 μg), oligo‐dT primer (Invitrogen, Carlsbad, CA, USA), dNTP, and reverse transcriptase (Superscript II, Invitrogen). qPCR was conducted with a cDNA template, 2× Power SYBR Green (Toyobo Co., Osaka, Japan), and specific primers (**Supplementary Table S1**) using the following cycle: 15 sec at 95°C, 30 sec at 55°C, and 60 sec at 70°C. Fluorescence intensities were determined at the end of the extension phase of each cycle. Values measured during the exponential phase of PCR amplification were used to define threshold cycles (Ct). The expressions of target genes were normalized versus β‐actin (housekeeping gene) based on Ct values at constant fluorescence intensity, as described by (25).

### 2.8 Western blot analysis

Liver total protein was extracted using Pro‐Prep Protein Extraction Solution (iNtRON Biotechnology, Seongnam, Korea), and quantified using a SMARTTM BCA Protein Assay Kit (Thermo Scientific). Total proteins (20-30 µg) were loaded and separated by 4–20% sodium dodecyl sulfate-polyacrylamide gel electrophoresis (SDS‐PAGE) for 2 h, after which resolved proteins were transferred to nitrocellulose membranes for 2 h at 40 V. Membranes were then incubated separately overnight at 4°C with specific primary antibodies (**Supplementary Table S2**). Probed membranes were then washed with washing buffer (137 mM NaCl, 2.7 mM KCl, 10 mM Na_2_HPO_4_, and 0.05% Tween 20) and incubated with 1:2,000 diluted horseradish peroxidase (HRP)‐conjugated goat anti‐rabbit IgG (Invitrogen) at RT for 1 h. Finally, the membrane blots were developed using Amersham ECL Select Western Blotting detection reagent (GE Healthcare, Little Chalfont, UK). Chemiluminescence signals originating from specific bands were detected using FluorChemi®FC2 (Alpha Innotech Co., San Leandro, CA, USA).

### 2.9 Statistical analysis

The statistical analyses were performed using GraphPad Prism 8.0 (GraphPad Software Inc., San Diego, CA, USA). The significance of intergroup differences was determined by one‐way analysis of variance (ANOVA) and Tukey’s *post hoc* t‐test for multiple comparisons. Unless otherwise specified, error bars represent SEMs. P values of <0.05 were considered statistically significant. Individual P values are provided in the figure legends. All experiments were performed twice independently.

## 3 Results

### 3.1 Chemical profile of MED

**Figure 1:**
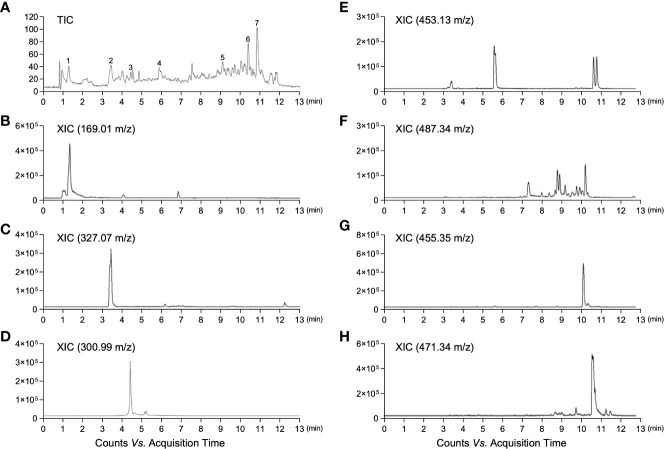
LC-ESI-QTOF-MS chromatograms of MED. **(A)** Total ion chromatogram (TIC) of MED obtained by LC-MS; **(B)** Gallic acid: extracted ion chromatogram (XIC) of m/z 169.01 in negative mode; **(C)** Bergenin: XIC of m/z 327.07 in negative mode; **(D)** Ellagic acid: XIC of m/z 300.99 in negative mode; **(E)** Viniferin: XIC of m/z 453.13 in negative mode; **(F)** Asiatic acid: XIC of m/z 487.34 in negative mode; **(G)** Oleanolic acid: XIC of m/z 455.35 in negative mode; and **(H)** Hydroxyursolic acid: XIC of m/z 471.34 in negative mode.

Firstly, we analyzed the distribution of the bioactive components in MED to predict its potential for anti-obesity activity. LC-ESI-MS analysis in the negative ion mode was performed to investigate the chemical profile of MED. A representative total ion chromatogram (TIC) of LC-ESI-MS is shown in **Figure 1A**. Among the various peaks of TIC, seven bioactive compounds were identified. In addition, it was confirmed through their extracted ion chromatogram (XIC) that each peak was identified as gallic acid, bergenin, ellagic acid, ϵ-viniferin, asiatic acid, oleanolic acid, and 2α-hydroxyursolic acid (**Figures 1B–H** and **Table S3**). These results show that MED is potentially used as an anti-obesity treatment.

### 3.2 Inhibitory effect of MED on obesity phenotypes in *Lep* KO mice

**Figure 2:**
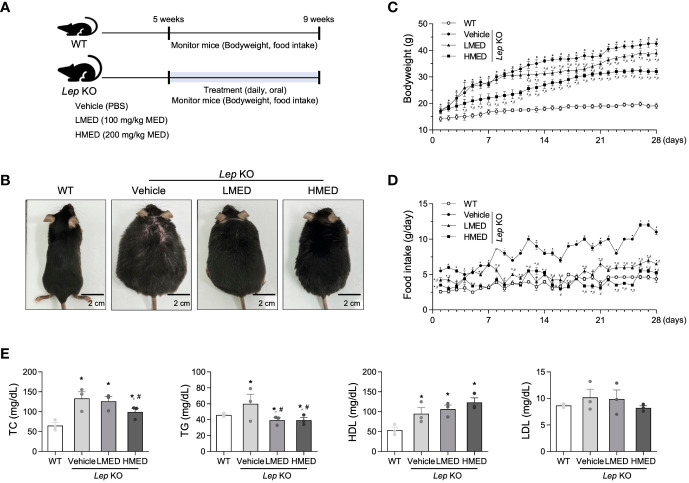
Body weights, food intakes, and serum lipid profiles of *Lep* KO mice treated with MED. **(A)** A schematic representation of the animal experiment. **(B)** Representative images of wild-type (WT) mice and *Lep* KO mice treated with 1x PBS (Vehicle), 100 mg/kg of MED (LMED), or 200 mg/kg of MED (HMED), n=7, size bar = 2 cm. The body weights **(C)** and daily food intakes **(D)** of WT mice and *Lep* KO mice treated with Vehicle, LMED, or HMED during the 4-week experiment. **(E)** TC, TG, HDL, LDL level analyses in the serum of WT mice and Lep KO mice treated with Vehicle, LMED, or HMED after 4-week experiment. Results are presented as means ± SEMs (n = 10). ^*^ *p* < 0.05 vs. the WT group; ^#^ *p* < 0.05 vs. the Vehicle-treated *Lep* KO group (*t*-test). TC, total cholesterol; TG, triglyceride; HDL, high density lipoprotein; LDL, low density lipoprotein.

The alterations in body weight, food intake, and serum lipid profiles were measured over the 4-week administration period of MED administration to determine whether MED could ameliorate obesity phenotypes in *Lep* KO mice (**Figure 2A**). A significant difference in body weight was observed between the WT and Vehicle-treated *Lep* KO groups. However, body weights were dose-dependently lower in the MED-administered groups than in the Vehicle-treated *Lep* KO group from experimental days 14 to 28. Body weight gains in the LMED and HMED groups were significantly lower (by 8.2% and 24.7%, respectively) than in the Vehicle-treated *Lep* KO group (**Figures 2B, C**). Moreover, food intake was higher in the Vehicle-treated *Lep* KO group than in the WT group during the experiment, and dramatically reduced dose-dependently after MED administration (**Figure 2D**).

Furthermore, TG and TC serum concentrations in the LMED and HMED groups were significantly lower than in the Vehicle-treated *Lep* KO group, although the concentrations of HDL-C were significantly higher (**Figure 2E**). These results demonstrate that MED treatment for 4 weeks can suppress body weight gain and improve serum TC, TG, and HDL-C levels.

### 3.3 Inhibitory effect of MED on fat accumulation in abdominal fat tissue

**Figure 3:**
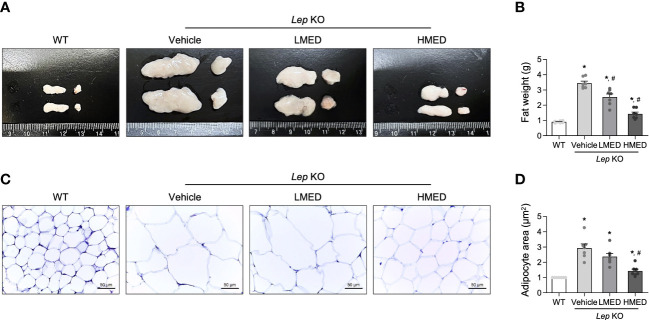
Weights and average areas of adipocytes in the fat tissues of *Lep* KO mice treated with MED. **(A)** Representative fat image showing epididymal (left) and retroperitoneal (right) fat harvested from the abdominal region of mice in the WT, Vehicle-treated *Lep* KO, LMED, and HMED groups. **(B)** Fat weights were calculated by adding the weights of epididymal and retroperitoneal fat. Fat tissues were collected from 7 mice per group, and fat tissue weights were measured in duplicate for each tissue type. **(C)** The representative image of H&E-stained epididymal fat tissues in all groups at 200×, size bar = 50 μm. **(D)** Plots of average adipocyte areas in epididymal fat tissues as determined using Image J. H&E-stained fat tissues of seven mice per group were examined, and adipocyte areas were measured in duplicate on each slide. Results are presented as means ± SEMs. ^*^ *p* < 0.05 vs. the WT group; ^#^ *p* < 0.05 vs. the Vehicle-treated *Lep* KO group (*t*-test).

Next, the effect of MED on abdominal fat tissue was investigated. Abdominal fat mass, including epididymal and retroperitoneal fat, was significantly (11.7-fold) more prevalent in the Vehicle-treated *Lep* KO group compared to WT controls (**Figures 3A, B**). The mean weights of abdominal fat in the LMED and HMED groups were 2.56 ± 0.25 g and 1.38 ± 0.44 g, respectively. This was significantly lower than the mean weight of the Vehicle-treated *Lep* KO group (3.02 ± 0.29 g). Also, the average area of adipocytes in H&E-stained fat tissues was significantly and dose-dependently lower in the LMED and HMED groups compared to the Vehicle-treated *Lep* KO group (**Figures 3C, D**). These results indicate that the suppressive effects of MED on obesity phenotypes are closely associated with the inhibition of fat accumulation in *Lep* KO mice.

### 3.4 Inhibitory effect of MED on hepatic steatosis in liver tissues of *Lep* KO mice

**Figure 4:**
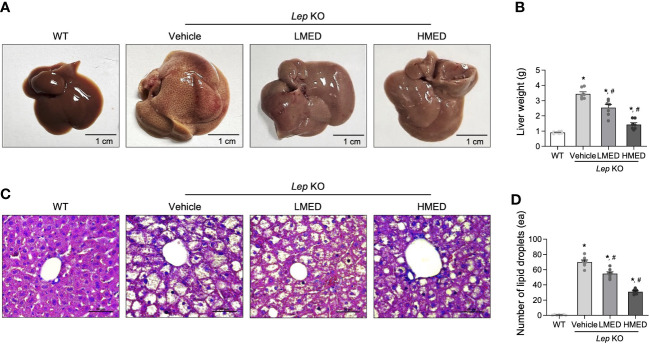
Hepatic lipid accumulation in *Lep* KO mice treated with MED. **(A)** Representative liver images of a WT and *Lep* KO mouse treated with Vehicle, LMED, or HMED, size bar = 1 cm. **(B)** Measurement of liver weights. Livers were collected from 7 mice per group and weights were measured twice. **(C)** Representative images of H&E stained liver sections, size bar = 50 μm. **(D)** Circular lipid droplets in H&E-stained sections reflected lipid accumulations. Total numbers of lipid droplets were measured in H&E-stained fat sections at 200× using the Leica Application Suite. Numbers of lipid droplets in H&E-stained tissues of 7 mice per group were counted in duplicate. Results are presented as means ± SEMs. ^*^ *p* < 0.05 vs. the WT group; ^#^ *p* < 0.05 vs. the Vehicle-treated *Lep* KO group (*t*-test).

The changes in liver weight and pathological characteristics were measured to investigate whether MED was associated with a reduction in hepatic steatosis. The liver weights of the Vehicle-treated *Lep* KO group were significantly greater (by 3.7-fold) than those of the WT group (**Figures 4A, B**). However, compared to the Vehicle-treated *Lep* KO group, MED supplementation dose-dependently reduced liver weights by 23.8% and 56.1% in the LMED and HMED groups, respectively. Furthermore, average numbers of lipid droplets in liver tissue sections (a surrogate of fat accumulation in the liver) showed a similar pattern of suppression. **Figures 4C, D** show that lipid drop numbers in liver tissue were significantly and dose-dependently lower in the LMED and HMED groups than in the Vehicle-treated KO group, although they were considerably higher in the Vehicle-treated *Lep* KO group than in the WT group. These findings suggest that the ameliorating effects of MED on obesity phenotypes are associated with the inhibition of hepatic steatosis in *Lep* KO mice.

### 3.5 Effect of MED on adipogenesis, lipogenesis, and lipolysis in liver tissue

**Figure 5:**
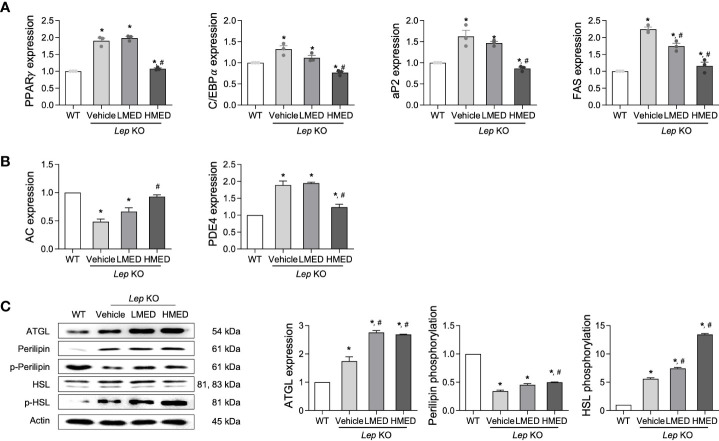
Expressions of proteins related to the lipid metabolism pathway in the liver tissues of *Lep* KO mice treated with MED. Expressions of lipogenesis-related genes. **(A)** Transcript levels of PPARγ, C/EBPα, aP2, and FAS were measured by RT-qPCR in WT mice and *Lep* KO mice treated with Vehicle, LMED, or HMED; and **(B)** Expression levels of lipogenesis-related genes. Transcript levels of AC and PDE4 were analyzed by using RT-qPCR in WT mice and Vehicle-treated *Lep* KO, LMED, and HMED mice. **(C)** Expression levels of proteins related to lipid metabolism. ATGL, perilipin p-perilipin, HSL, and p-HSL were detected using specific antibodies, and actin was used as the internal control. Results are presented as means ± SEM (n=5). ^*^ *p* < 0.05 vs. the WT group; ^#^ *p* < 0.05 vs. the Vehicle-treated *Lep* KO group (*t*-test).

Subsequently, we examined whether the inhibitory properties of MED on hepatic steatosis could be accompanied by an alteration in lipid metabolism. Four adipogenesis and lipogenesis-related genes (PPARα, C/EBPα, aP2, and FAS) had higher mRNA levels in the liver of the Vehicle-treated *Lep* KO group than in the WT group. However, MED-treated groups showed significant and dose-dependent decreases in the mRNA levels of PPARα, C/EBPα, aP2, and FAS in the liver tissue (**Figure 5A**). Moreover, MED had similar effects on lipolysis. The mRNA levels of two genes, AC and PDE4 (both positively associated with lipolysis), were analyzed using specific primers in the liver tissue. The mRNA levels of AC in liver tissues were greater in the MED-treated groups than in the Vehicle-treated KO group, although levels remained higher than in the WT group. Interestingly, PDE4 mRNA levels exhibited the opposite pattern (**Figure 5B**). In addition, the increases in AC mRNA levels observed in the liver tissue of MED-treated mice matched those of three lipogenic proteins in these mice. MED-treated mice had dose-dependently higher levels of ATGL mRNA expression than in the WT group. Additionally, MED increased the phosphorylation of HSL and perilipin in the liver, but at lower levels than in the WT group (**Figure 5C**). Consequently, the findings suggest that the inhibitory effects of MED on hepatic steatosis are associated with the inhibition of adipogenesis and lipogenesis and the stimulation of lipolysis in the livers of *Lep* KO mice.

### 3.6 Inhibitory effect of MED on hepatic inflammation

**Figure 6:**
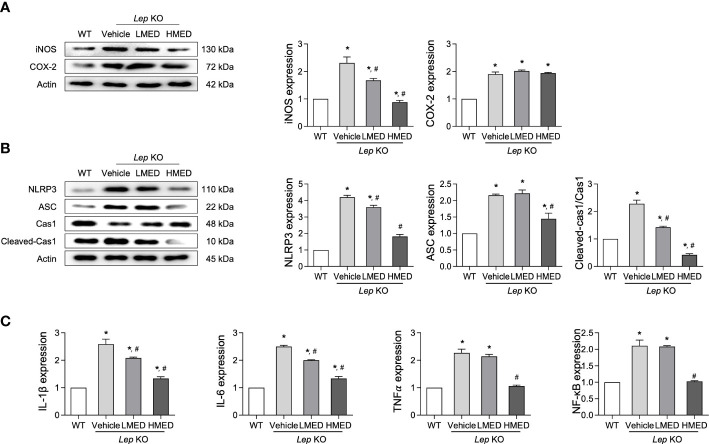
Expressions of inflammatory regulators in the liver tissues of MED-treated *Lep* KO mice. **(A)** Expression levels of proteins in the iNOS-mediated COX-2 induction pathway. Levels of iNOS and COX-2 proteins were detected in the liver tissues of WT and MED-treated *Lep* KO mice using specific antibodies. **(B)** Expression levels of proteins in the NLRP3 inflammasome pathway. Levels of the NLRP3, ASC, Cas-1, and cleaved Cas-1 were detected in the liver tissues of WT and *Lep* KO mice treated with MED using specific antibodies. Actin was used as the internal control. **(C)** Transcriptional levels of inflammatory cytokines. Levels of IL-1β, IL-6, TNF-α, and NF-κB mRNA were detected in the liver tissues of WT and MED-treated *Lep* KO mice using specific primers. Results are presented as means ± SEMs (n=5). ^*^ *p* < 0.05 vs. the WT group; ^#^ *p* < 0.05 vs. the Vehicle-treated *Lep* KO group (*t*-test).

Hepatic steatosis can be accompanied by an alteration in inflammation (26). We examined the expression level of key proteins in the iNOS-induced COX-2 mediated pathway and the NLRP3 inflammasome pathway, in addition to the transcription level of inflammatory cytokines in liver tissue after the administration of MED. The protein levels of iNOS and COX-2 were higher in the liver tissues of Vehicle-treated *Lep* KO mice than in WT controls. **Figure 6A** shows that MED administration reduced both and dose-dependently reduced iNOS expression. Furthermore, the inhibitory effects of MED were detected in the NLRP3 inflammasome pathway, which includes NLRP3 (NLR family pyrin domain containing 3), ASC (apoptosis-associated speck-like protein containing a CARD), and cleaved Cas-1. The protein levels of NLRP3, ASC, and cleaved Cas-1 were remarkably higher in Vehicle-treated *Lep* KO mice than in WT controls, but they were significantly lower in *Lep* KO mice treated with MED than in Vehicle-treated *Lep* KO mice (**Figure 6B**). Furthermore, MED had a similar inhibitory effect on the mRNA levels of inflammatory-related cytokines, and IL-1β, IL-6, TNF-α, and NF-κB mRNA levels were higher in the Vehicle-treated *Lep* KO group than in the WT group. Moreover, the administration of MED significantly decreased the expressions of these cytokine levels compared to the Vehicle-treated KO group (**Figure 6C**). These results suggest that the inhibitory effects of MED on hepatic steatosis are associated with the suppression of the inflammatory response through the downregulations of the iNOS-COX-2 pathway, the NLRP3 inflammasome pathway, and the expression of inflammatory cytokines.

### 3.7 Stimulatory effects of MED on β-oxidation in liver tissue

**Figure 7:**
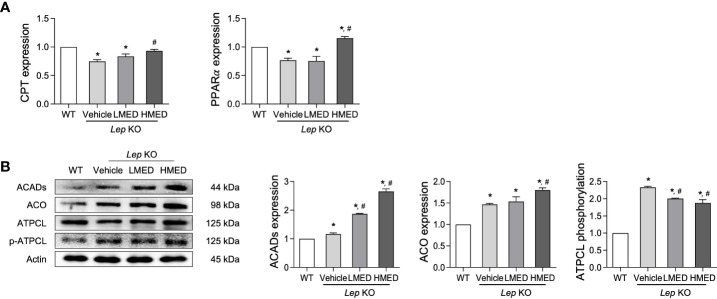
Levels of the β-oxidation regulators in the liver tissues of *Lep* KO mice treated with MED. **(A)** Levels of transcription factors. Levels of CPT and PPARγ mRNAs were assessed in the liver tissues of WT and *Lep* KO mice treated with MED using specific primers. **(B)** Expression levels of β-oxidation enzymes. Levels of ACADs, ACO, ATPCL, and p-ATPCL proteins were detected in the liver tissues of WT and *Lep* KO mice treated with MED using specific antibodies. Actin was used as the internal control. Results are presented as means ± SEMs (n = 5). ^*^ *p* < 0.05 vs. the WT group; ^#^ *p* < 0.05 vs. the Vehicle-treated *Lep* KO group (*t*-test).

Finally, we explored whether the inhibition effects of MED on hepatic steatosis can be accompanied with stimulation of β-oxidation. The level of β-oxidation-related factors was analyzed in the liver of *Lep* KO mice after treatment with MED. The mRNA levels of PPARα and CPT in the liver tissue were significantly lower in Vehicle-treated KO mice than in WT controls. MED treatment dose-dependently increased these mRNA levels (**Figure 7A**). In addition, significant increases were detected in protein levels of two β-oxidation-related proteins (ACADs and ACO) in the liver tissue after MED treatment. However, MED administration dose-dependently reduced ATPCL phosphorylation in the liver tissue (**Figure 7B**). These results demonstrate that the inhibitory effects of MED on hepatic steatosis are associated with enhanced β-oxidation via the regulations of CPT and PPARα.

## 4 Discussion

Over the past half-century, tremendous progress has been made in managing metabolic diseases closely related to obesity. However, anti-obesity medications have questionable safety and often prove ineffective (8). Recently, several studies have focused on discovering natural compounds and extracts with disease-preventing or health-promoting effects (20). Some bioactive components with few side effects have been investigated for their anti-obesity effects and the mechanisms responsible (20, 27–31). *D. tuberculatus* has attracted considerable interest as a potential treatment for a number of diseases, particularly those associated with inflammation and anti-photoaging. We were interested in providing scientific evidence on the anti-obesity effects of MED, as considerations of lipid metabolism and related-inflammatory responses are crucial for treating obesity. In this study, we found that the primary therapeutic effects of MED are inhibiting body weight gain, food intake, liver weight, fat accumulation, lipid metabolism, inflammation, and the stimulation of β-oxidation in *Lep* KO mice.

Body weight gain, fat tissue, and serum lipid profile can be crucial indicators for assessing the anti-obesity effect (32). Oral administration of MED (100 and 200 mg/kg, daily) for four weeks reduced body weight gain, fat weight, and adipocyte area in a dose-dependent manner. The increased levels of serum TC and TG in *Lep* KO mice were effectively inhibited after MED administration. Although no significant change was detected in LDL-C levels, our results are consistent with previously reported results of anti-obesity studies. Furthermore, the MED-treated group exhibited a reduction in food intake comparable to that of the Vehicle group in *Lep* KO mice, showing similar to the WT group. However, we did not observe any indications that the reduced food intake was due to the unpalatable nature of MED. There were no noticeable differences in the appearance or behavior of the MED-treated mice compared to the WT control group, and the MED-treated mice showed no signs of aversion to the MED.

The liver is the most important organ in lipid metabolism, which involves lipid absorption, synthesis, transportation, and degradation. Fat accumulates in hepatocytes during obesity-induced lipid metabolic disorders, and the number of lipid droplets in the liver increases, leading to hepatocellular steatosis, a severe pathologic feature in the obese (33). These pathological features were observed in the present study, and in particular, many fat droplets accumulated in the liver acini of *Lep* KO mice. In our study, oral administration of MED reduced liver steatosis, as determined by liver weights and histological findings, and the number of lipid droplets was significantly reduced in the livers of *Lep* KO mice.

Obesity results from an imbalance between lipogenesis and lipolysis (34). Adipose tissues store energy as TGs within lipid droplets formed by lipogenesis, and fatty acids (FAs) are released from these stores via lipolysis. However, both processes are significantly elevated in obese patients (35). The regulatory effects of several herbs on lipogenesis and lipolysis have been investigated. *Garcinia* (36) and *Citrus depressa Hayata* (37) inhibited lipogenesis by downregulating lipogenesis-related genes. *Salix matsudana leaves* (38), *Actinidia arguta* roots (39), and *Zicao* roots (40) have been reported to induce lipolysis in adipocytes. The rhizomes of Curcuma longa were found to affect both mechanisms, specifically, they suppressed lipogenesis and increased lipolysis by upregulating lipases like adipose TGs, lipase, hormone-sensitive lipase, adiponectin, and AMP-activated protein kinase (41). In addition, the roots and stems of *Salacia reticulate* upregulated lipogenesis genes and downregulated lipolysis genes through AMPkα activation in adipocytes (42). Adipogenesis is a process for the differentiation of preadipocytes into mature adipocytes (lipid-accumulating and insulin-responsive adipocytes) and is regulated by adipogenic transcription factors (PPARγ and C/EBPα) (43, 44). When these factors are activated, lipogenesis is induced by lipogenic genes (aP2 and FAS) and the maintenance of adipocyte phenotypes during the late adipocyte differentiation (45, 46). In our study, MED administration regulated adipogenesis and lipogenesis by suppressing related transcriptional factors and enhanced the expression of lipolytic proteins in *Lep* KO mice. The results obtained for the effects of MED on lipogenesis and lipolysis are similar to those of previous studies that investigated the lipogenesis-inhibiting and lipolysis-stimulating effects of natural products.

Inflammatory responses can damage hepatocytes because proinflammatory cytokines can cause adipocyte hypertrophy and adipose tissue disorders (26), and thus regulators of inflammatory responses are considered targets of anti-obesity therapy (47). In particular, NLRP3 inflammasome is activated by cellular stress response and obesity-induced inflammatory response in adipocytes and promotes the autoactivation of Cas-1, which induces the activations of mediators of immune response (48, 49). Also, IL-1β stimulates the secretions of IL-6 and TNF-α to regulate the migration and infiltration of immune cells, while IL-18 promotes the recruitment and activation of immune cells (48, 50). Interestingly, adipose tissue-derived inflammation in a high-fat diet-induced model of obesity was effectively suppressed by several natural products (29–31). Representative, Mulberry leaf and fruit extract significantly decreased hepatic levels of TNF-α and IL-1β, though iNOS levels were not significantly suppressed (29). Also, another natural product, *Malus hupehensis* administration, promotes reducing serum levels of TNF-α, IFN-γ, IL-1β, and IL-6 (31). MED suppressed the transcript levels of the proinflammatory cytokines IL-1β, IL-6, TNF-α, and NF-κB and also reduced the mRNA expressions of three inflammasome regulators in the livers of *Lep* KO mice. Our results provide the first evidence that MED acts by targeting obesity-induced inflammatory response in *Lep* KO mice.

The changes in β-oxidation-related protein levels provide a method for evaluating the anti-obesity effects of natural products. The β-oxidation of FAs produces energy by breaking them down into CO_2_ and ketone bodies. A number of several enzymes, including acyl-CoA oxidase-1 (ACO-1), carnitine palmitoyltransferase-1 (CPT-1), and acyl-CoA dehydrogenases (ACADs), which are expressional regulated by PPARα, are involved in this process (51). According to reports, several natural products stimulate the β-oxidation of FAs in high fat diet-induced obese animal models (30, 31). The water-soluble extract of *Cucurbita moschata* was found to increase ACO-1 and CPT-1 by upregulating PPARα (30), while the treatment of Mulberry leaf and fruit extracts was reported to significantly upregulate PPARα and CPT1 mRNA expression in liver (29). In the present study, MED increased the protein expressions of ACADs and ACO in *Lep* KO mice by upregulating the mRNA levels of PPARα. The effects of MED on β-oxidation stimulation are similar to those previously reported for other natural products. Additionally, our findings indicate that MED acts as a β-oxidation stimulator.

Even though the anti-obesity effects of MED are proved in various targets in this study, since MED is a mixture of complex compounds, it is hard to explain the mechanism of its action on adipose tissue clearly. According to the chemical profile of MED, gallic acid, bergenin, ellagic acid, ϵ-viniferin, asiatic acid, oleanolic acid, and 2α-hydroxyursolic acid are regarded as the main components, and these individual components have been reported to show anti-obesity effects. Gallic acid decreased body weights and adipose tissue weights of peritoneal and epididymal tissues in addition to the serum TAG, phospholipid, total cholesterol, and LDL-cholesterol in high-fat diet-fed rats (52). Bergenin, olanolic acid, and ϵ-viniferin also lowered body weight gain and the weight of adipose tissue in obese mice (53–55). Moreover, asiatic acid attenuated body weight gain, tissue lipids, mRNA levels of PPAR γ, FAS, aP2, and inflammatory factor TNF-α in HFD-fed rats (56). Considering that all of these components are included in MED, MED has a high potential to be a novel treatment for obesity.

## 5 Conclusions

This study was performed to determine the therapeutic effects of MED on obesity using *Lep* KO mice. MED-induced alterations in body weights, food intakes, serum lipid profiles, and lipid accumulation were analyzed in *Lep* KO mice after treatment for 4 weeks. In addition, we investigated changes in the expression of genes involved in lipid accumulation, inflammation, and β-oxidation. Our results provide scientific evidence that MED has considerable potential to prevent obesity and ameliorate its effects.

## Data availability statement

The original contributions presented in the study are included in the article/**Supplementary Material**. Further inquiries can be directed to the corresponding authors.

## Ethics statement

The animal study was reviewed and approved by Institutional Animal Care and Use Committee of Pusan National University (PNU-IACUC).

## Author contributions

Conceptualization, DH. Methodology, DH and SC. Software, SL. Validation, SL. Formal analysis, SL. Investigation, SL, YJ, JK, YR, AS, HS, JP and SP. Resources, BD and OS. Data curation, SL and YJ. Writing and original draft preparation, DH. Writing, reviewing, and editing, SC. Visualization, DH and SC. Supervision, DH. Project administration, DH. Funding acquisition, DH. All authors contributed to the article and approved the submitted version.
