# Supplementary material for: Dipterocarpus tuberculatus as a promising anti-obesity treatment in Lep knockout mice
Source: Front Endocrinol (Lausanne). 2023 May 26;14:1167285. doi: 10.3389/fendo.2023.1167285 (PMC10273273; doi:10.3389/fendo.2023.1167285)
Supplement: Supplementary file 1 [file DataSheet_1.pdf]

Supplementary Material

Dipterocarpus tuberculatus as a promising anti-obesity treatment in *Lep* knockout mice

Yu Jeong Roh[1,#], Su Jin Lee[1,#], Ji Eun Kim[1], You Jeong Jin[1], Ayun Seol[1], Hee Jin Song[1], Jumin Park[2], So Hae Park[1], Bounleuane Douangdeuane[3], Onevilay Souliya[3], Sun Il Choi[4*], and Dae Youn Hwang[1,5,*]

*** Correspondence:** Dae Youn Hwang: dyhwang@pusan.ac.kr, Sun Il Choi: sunil.choi@hotmail.com

1 Supplementary Tables

Table S1. Primer sequences for RT-PCR analyses

Primer name	Sequence (from 5' to 3')	Product size (bp)
PPARγ		
Forward	GAG TTC ATG CTT GTG AAG GAT GCA AGG	80
Reverse	CAT ACT CTG TGA TCT CTT GCA CG	
C/EBPα		
Forward	GTG GAC AAG AAC AGC AAC GAG TAC	71
Reverse	GGA ATC TCC TAG TCC TGG CTT GC	
FAS		
Forward	GAT CCT GGA ACG AGA ACA CGA TCT GG	128
Reverse	AGA CTG TGG AAC ACG GTG GTG GAA CC	
aP2		
Forward	GAA CCT GGA. AGC TTG TCT CCA GTG	72
Reverse	GAT GCT CTT CAC CTT CCT GTC GTC TGC	
Adenylyl Cyclase		
Forward	CGG AGG TTG CTG CAT AAC ATT	106
Reverse	ACA CAT TCA CAC GAC TGG TAG TAC AG	
PDE4		
Forward	TTC CCT CAT CAC CCT ACC TAT CA	98
Reverse	GGC AGA GCA GAG GTT CAA GCT	
CPT1		
Forward	GGC AGA GCA GAG GTT CAA GCT	81
Reverse	GCC AGC GCC CGT CAT	
PPARα		
Forward	TGG CAA AAG GCA AGG AGA AG	104
Reverse	CCC TCT ACA TAG AAC TGC AAG GTT T	
NF-κB		
Forward	GTA ACA GCA GGA CCC AAG GA	501
Reverse	AGC CCC TAA TAC ACG CCT CT	
TNF-α		
Forward	CCT GTA GCC CAC GTC GTA GC	374
Reverse	TTG ACC TCA GCG CTG ACT TG	
IL-6		
Forward	TTG GGA CTG ATG TTG TTG ACA	200
Reverse	TCA TCG CTG TTG ATA CAA TCA GA	
IL-1β		
Forward	CAG TTC TGC CAT TGA CCA	218
Reverse	TCT CAC TGA A AC TCA GCC GT	
β-Actin		
Forward	TGG AAT CCT GTG GCA TCC ATG AAA C	349
Reverse	TAA AAC GCA GCT CAG TAA CAG TCC G	

Table S2. Antibody list for western blot analyses

Antibody name	Company (City, Country)	Dilution ratio
Anti-perilipin antibody	Cell Signaling Technology (Danvers, MA, USA)	1:1,000
Anti-p-perilipin antibody	Cell Signaling Technology (Danvers, MA, USA)	1:1,000
Anti-HSL antibody	Cell Signaling Technology (Danvers, MA, USA)	1:1,000
Anti-p-HSL antibody	Cell Signaling Technology (Danvers, MA, USA)	1:1,000
Anti-ATGL antibody	Cell Signaling Technology (Danvers, MA, USA)	1:1,000
Anti-ACADs	Abcam (Cambridge, UK)	1:1,000
Anti-ACO1	Abcam (Cambridge, UK)	1:1,000
Anti-ATP-Citrate Lyase	Cell Signaling Technology (Danvers, MA, USA)	1:1,000
Anti-p-ATP-Citrate Lyase	Cell Signaling Technology (Danvers, MA, USA)	1:1,000
Anti-iNOS	Thermo Fisher Scientific Co. Ltd. (Waltham, MA, USA)	1:1,000
Anti-COX2	Cell Signaling Technology (Danvers, MA, USA)	1:1,000
Anti-NLRP3 antibody	Cell Signaling Technology (Danvers, MA, USA)	1:1,000
Anti-ASC antibody	Cell Signaling Technology (Danvers, MA, USA)	1:1,000
Anti-Caspase-1 antibody	Cell Signaling Technology (Danvers, MA, USA)	1:1,000
Anti-β-actin antibody	Cell Signaling Technology (Danvers, MA, USA)	1:1,000

Table S3. LC-ESI-QTOF-MS chromatograms of the methanolic extract of MED.

**Compound name**	**Molecular formula**	**Molecular weight (g/mol)**	**Time (min)**	**Expected *m/z***	**Polarity**	**Observed *m/z***	
Gallic acid	$C_7H_6O_5$	170.12	1.343	169.0142	$[M-H]^-$	169.0140	(Figure 1B)
Bergenin	$C_{14}H_{16}O_9$	328.27	3.475	327.0722	$[M-H]^-$	327.0725	(Figure 1C)
Ellagic acid	$C_{14}H_6O_8$	302.19	4.466	300.9990	$[M-H]^-$	300.9993	(Figure 1D)
ε-Viniferin	$C_{28}H_{22}O_6$	454.47	5.878	453.1344	$[M-H]^-$	453.1350	(Figure 1E)
Asiatic acid	$C_{30}H_{48}O_5$	488.70	9.089	487.3429	$[M-H]^-$	487.3427	(Figure 1F)
Oleanolic acid	$C_{30}H_{48}O_3$	456.70	10.374	455.3531	$[M-H]^-$	455.3519	(Figure 1G)
2α-Hydroxyursolic acid	$C_{30}H_{48}O_4$	472.70	10.850	471.3480	$[M-H]^-$	471.3475	(Figure 1H)
